# Translational development and first-in-human compassionate infusion of NK-92 cells expressing a CD5-based chimeric antigen receptor (SRCD5CAR-NK-92) in a patient with multidrug-resistant fusariosis

**DOI:** 10.3389/fimmu.2026.1772830

**Published:** 2026-05-08

**Authors:** María Velasco-de-Andrés, Pedro Puerta-Alcalde, Jiri Eitler, Lydia Krutz, Mariana Chumbita, Marta Español-Rego, Cristina Català, Laura Carrillo-Serradell, Violeta Planells-Romeo, Lucía Aragón-Serrano, Andrea Vergara, Amanda Isabel Pérez-Valencia, Patricia Monzó-Gallo, Antonio Gallardo-Pizarro, María Teresa Cibeira, Alexandru Vlagea, José Luis Caro, Eduard Palou, María Suárez-Lledó, Alex Soriano, Gonzalo Calvo, Álvaro Urbano-Ispizua, Torsten Tonn, Carolina Garcia-Vidal, Francisco Lozano

**Affiliations:** 1 Immunoreceptors del sistema innat i adaptatiu, Institut d’Investigacions Biomèdiques August Pi i Sunyer (IDIBAPS), Barcelona, Spain; 2 Department de Malalties Infeccioses, Hospital Clínic de Barcelona, Barcelona, Spain; 3 Departament de Medicina, Universitat de Barcelona (UB), Barcelona, Spain; 4 Experimental Transfusion Medicine, Faculty of Medicine Carl Gustav Carus, Dresden University of Technology, Dresden, Germany; 5 Institute for Transfusion Medicine Dresden, German Red Cross Blood Donation Service North-East, Dresden, Germany; 6 German Cancer Consortium (DKTK), Dresden, Germany; 7 Servei d’Immunologia, Centre de Diagnòstic Biomèdic, Hospital Clínic de Barcelona, Barcelona, Spain; 8 Servei de Microbiologia i Parasitologia-CDB, Hospital Clínic de, Barcelona, Spain; 9 Institut de Salut Global (ISGlobal), Barcelona, Spain; 10 Departament de Fonaments Clínics, University of Barcelona, Barcelona, Spain; 11 CIBER Enfermedades Infecciosas (CIBERINFEC), Madrid, Spain; 12 Servei de Hematologia, Hospital Clínic de Barcelona, Barcelona, Spain; 13 Servei de Farmacologia Clínica, Area Medicament, Hospital Clínic de Barcelona, Barcelona, Spain; 14 Clinical Pharmacology, Institut d’Investigacions Biomediques August Pi i Sunyer (IDIBAPS), Barcelona, Spain; 15 Departament de Biomedicina, Universitat de Barcelona (UB), Barcelona, Spain

**Keywords:** adoptive cell therapy, CAR, CD5, fusariosis, multidrug-resistance, NK-92 cells

## Abstract

**Introduction:**

Invasive fungal infections (IFI) remain a major therapeutic challenge due to limited antifungal options, emergence of multidrug-resistant (MDR) strains and high mortality rates. This has turned up the development of immune-based therapies to restore and/or enhance anti-fungal immune responses into a clinical priority. This is in line with the recent demonstration that antifungal activity of primary (cord blood-derived) NK cells can be potentiated by endowing them with a CD5-based second-generation chimeric antigen receptor (SRCD5CAR) targeting β-glucans, a constitutive and broadly distributed component of fungal cell walls. Building on this, we aimed at exploring whether the reported constitutive antifungal activity of leukemia-derived NK-92 cells can also be potentiated by engineering them for further potential use as a homogeneous and ready-to-use source of allogeneic cells for adoptive transfer therapy in IFI.

**Methods:**

NK-92 cells lentivirally transduced and selected for stable and high-level expression of the SRCD5CAR for further testing in in vitro fungal killing assays, as well as in an *in vivo* model of fungal infection.

**Results:**

SRCD5CAR-NK-92 cells showed superior antifungal activity than untransduced NK-92 cell counterparts. SRCD5CAR-NK-92 cells also exhibited superior activity against a MDR *Fusarium petroliphilum* isolate from a patient undergoing a hematological malignancy and devoid of alternative therapeutic options. Following compassionate use approval, the patient received escalating intravenous infusions of irradiated SRCD5CAR-NK-92 cells at 2-5 day intervals, without significant local or systemic adverse effects (e.g., cytokine storm or alloreactivity).

**Discussion:**

Despite the patient ultimately succumbed due to progression of the underlying hematological malignancy, this represents first-in-human use of SRCD5CAR-NK-92 cells in the context of a severe MDR IFI. The results highlight its potential as a safe and off-the-shelf therapeutic strategy, while underscoring the need for further investigation on efficacy and long-term outcomes.

## Introduction

Invasive fungal infections (IFI) cause substantial morbidity and mortality especially in immunocompromised patients (1). The emergence of rare, aggressive, and often multidrug-resistant (MDR) fungal strains that associate with poor prognosis, are forcing a change in IFI clinical management (2) currently limited to the scarce and significantly toxic therapeutic arsenal available. Consequently, there is an urgent need for innovative therapeutic approaches as recognized by the WHO’s 2022 first fungal priority pathogen list (FPPL), that points at *Candida albicans*, *Cryptococcus neoforman*s, *Aspergillus fumigatus* and *Fusarium* spp., as critical and high immediate priorities (3).

The close relationship between IFI and immunocompromised settings offers an opportunity for immune-based therapies to restore and/or enhance the innate and/or adaptive anti-fungal immune responses in patients with IFI (4, 5). The current immunotherapeutic arsenal includes monoclonal antibodies (mAb), cytokines, vaccines and adoptive transfer of immune cells (6). This last option is supported by a single-center clinical study of two decades ago, in which *Aspergillus-*specific T cells were adoptively transferred to hematopoietic stem cell transplant (HSCT) patients with invasive aspergillosis (7). The complex, time-consuming and costly process of obtaining fungus-specific T-cells hindered further applications of this clinical strategy. These limitations can be partially solved by using autologous T cells engineered to express chimeric antigen receptors (CAR) against specific structural components of fungal cell surfaces (8–14), even if time and potential side effect (e.g., cytokine release syndrome and neurotoxicity) continue to pose clinical constraints. These can be overcome by the use of allogeneic NK cells, which hold promise for off-the-shelf CAR-NK therapies (15) as the involvement of both primary and leukemia-derived NK cells (i.e., NK-92 cells) in the innate antifungal response has now gained substantial evidence (16, 17). The feasibility of this strategy has been supported by recent evidence demonstrating the therapeutic potential of CAR-engineered NK-92 cells targeting mannan (18).

We have recently reported that the antifungal activity of primary (cord blood-derived) NK cells can be greatly potentiated by expressing a second-generation CD5-based CAR (SRCD5CAR) (19). Instead of using a fungal-specific single change variable fragment (scFv), the fungal-recognition domain of that CAR corresponds to the whole extracellular region of human CD5, a lymphocytic scavenger receptor with high binding affinity (*K*_d_ = 3.7 ± 0.2 nM) for β-glucans, which are constitutive and broadly-distributed component of fungal cell walls, which is absent from human cells (20).

Herein we report the development and functional assessment of NK-92 cells engineered and selected for stable and high-level surface expression of the SRCD5CAR as a potential homogeneous, unlimited and ready-to-use source of allogeneic cells for adoptive transfer therapy against IFI. This was supported by *in vitro* and/or *in vivo* pre-clinical experimental models of fungal infection, as well as by compassionate use authorization of SRCD5CAR-NK-92 cell infusion in a hematological patient undergoing a fungal infection by a MDR *F. petroliphilum*.

## Materials and methods

### Generation and selection of NK-92 cells stably expressing high surface levels of the SRCD5CAR construct

The lentiviral vector coding for the second-generation SRCD5CAR construct was generated by replacing the CD19-scFv coding sequence from the third-generation lentiviral vector pCCLsinPPT_EF1a_CART19 (21–23) with a *Mlu*I-*BspE*I restricted cDNA fragment from the human CD5 lymphocyte receptor. The resulting construct was under the transcriptional control of the EF1α promoter and contains the coding sequences for the human CD8α signal peptide, the whole extracellular region of CD5 (from R25 to D369), the human CD8α hinge and transmembrane region, and the cytoplasmic activating motifs of the human 4-1BB/CD137 and CD3ζ receptors.

Whole lentiviral particles were produced in HEK293T cells co-transfected with the psPAX2 and pMD2.G packaging vectors (both from Addgene) by using polyethyleneimine (PEI). Cell supernatants were harvested after 48 h and lentivirus were concentrated with PEG-it solution (System Biosciences) according to the manufacturer’s instructions.

As the source of NK-92 cells the sample used had been approved in earlier clinical trials (24, 25) and was provided to Torsten Tonn by H.G. Klingemann in 1997 (at the time Terry Fox Laboratory, BC Cancer Agency, Vancouver Canada) (26), and the SRCD5CAR- and GFP-expressing variants were cultured in X-VIVO 10 media (Lonza) containing 5% heat-inactivated human AB plasma (German Red Cross Blood Donation Service North-East, Dresden, Germany), and 500 IU/mL IL-2 (Proleukin; Novartis Pharma). NK-92 cells were lentivirally transduced at MOI 5 by 90 min spinoculation at 1000 x g in the presence of 8 µg/ml Polybrene (Sigma-Aldrich) to generate SRCD5CAR-NK-92 cells. Control GFP-NK-92 cells were generated by lentiviral transduction using the pSIEW-GFP vector, as previously described (27). SRCD5CAR-positive NK-92 cells were immunomagnetically enriched according to the manufacturer’s protocol using a biotinylated anti-CD5 antibody (clone UCHT2), all from Miltenyi Biotec. GFP-positive NK-92 cells were sorted by flow cytometry using a BD FACSAria II (BD Biosciences). The enriched and sorted cells yielded >95% purity of SRCD5CAR-NK-92 or GFP-NK-92 cells. The SRCD5CAR expression was tested regularly by flow cytometry with a PerCP Cy5.5-labeled anti-CD5 antibody (clone UCHT2; BD Pharmingen) and a BD FACSCanto II flow cytometer equipped with FlowJo software version 9 (BD Biosciences).

For patient’s infusion purposes SRCD5CAR-NK-92 cells were suspended in sterile saline solution plus 2.5% human albumin (Albuplan^®^, Grifols).

### *In vitro* assays of antifungal activity

*C. albicans* (SC5314; ATCC MYA‐2876) and *C. neoformans* var *grubii* (serotype A, H99; ATCC 208821) strains were kindly provided by Dr. Óscar Zaragoza (Centro Nacional de Microbiología, Instituto de Salud Carlos IIII, Majadahonda, Spain). Conidia (1 x 10^5^ CFU/mL) were co-cultured at different effector:target (E:T) ratios with SRCD5CAR-NK-92 and GFP-NK-92 cells for 4 h at 37 °C in a 5% CO_2_ atmosphere. After osmotic lysis of NK-92 cells, supernatants were seeded onto Sabouraud dextrose agar plates (Condalab) for 48 h at 30 °C for colony forming units (CFU) assessment. Killing percentage was calculated with regard to fungal cells cultured alone.

The antifungal activity of SRCD5CAR-NK-92 and GFP-NK-92 cells against *F. petroliphilum* was measured as the fungal’s metabolic activity using the XTT Cell Proliferation Assay Kit (CA031, Canxav) (17). Briefly, upon 18 h co-culture with *F. petroliphilum* at different E:T ratios (10:1, 1:1 and 1:10), NK-92 cells were lysed by 2 washes with sterile distilled water. Remaining fungal cells were incubated in XTT solution at 37 °C for 18 h and the absorbance of the supernatant measured at 450 nm, and 630 nm used as reference in a Tecan infinite 200Pro. The percentage of metabolic activity was calculated regarding fungal cells cultured alone.

The levels of human IFN-γ, perforin, CCL3, and CCL5 in co-culture supernatants were assessed by ELISA kits (BD OptEIA™ Human ELISA Set, Abcam, or R&D DuoSet ELISA Development System) following the manufacturer’s instructions.

### *In vivo* assessment of antifungal activity

Eight- to 10-week-old immune deficient NSG mice (NOD-scid IL-2Rγnull; Charles River Laboratories, France) were infected i.v. (tail vein) with *C. albicans* (3 x 10^3^ CFU/mouse) and 24 h later treated with a single i.v. infusion of SRCD5CAR-NK-92 or GFP-NK-92 cells (2 x 10^6^ cells/mouse). Mouse survival and body weight loss were monitored daily for 20 days. For kidney fungal load assessment mice were euthanized at 48 h post infection in parallel experiments. To this end, kidneys were aseptically removed and homogenized in PBS (3 mL) through 40 µm cell strainers (Biologix) and serial dilutions of the homogenates plated onto sabouraud dextrose agar plates (Condalab) for CFU/gr assessment after 48 h of incubation at 30 °C. All experimental mouse procedures were approved by the Animal Experimentation Ethical Committee of the University of Barcelona (CEEA 132/19 and 189/23).

### Patient case

**Figure 1:**
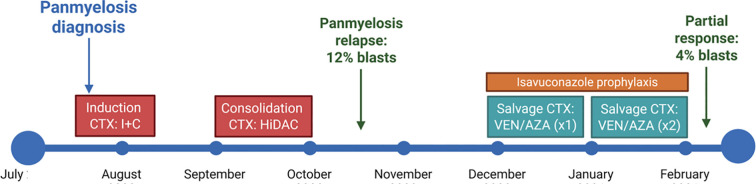
Timeline of patient’s previous hematological treatments. CTX, chemotherapy; I+C, idarubicin and cytarabine; VEN, venetoclax; AZA, azacytidine.

A 59-year-old patient attended at *Hospital Clinic de Barcelona*, Spain, was diagnosed with acute panmyelosis with myelofibrosis, and received induction chemotherapy with idarubicin and high-dose cytarabine, achieving a complete response. This was followed by consolidation therapy with high dose cytarabine. After the first consolidation cycle, the patient relapsed and salvage therapy with venetoclax plus azacytidine was initiated. Following two cycles, a partial response was observed, with a subsequent bone marrow aspirate showing 4% blasts (CD34++, CD33+, CD13+, CD117+, HLADR+, MPO-). Given the patient’s disease status and the availability of a matched unrelated donor, sequential allogeneic hematopoietic stem cell transplantation (allo-HSCT) was planned to be performed one month after confirmation of response. Primary antifungal prophylaxis with isavuconazole was initiated due to profound and prolonged neutropenia. While awaiting allo-HSCT, the patient developed a systemic fungal infection (skin and lung involvement) caused by a MDR *Fusarium petroliphilum*, and liposomal amphotericin B was initiated as targeted antifungal therapy (**Figure 1**). See clinical outcome and diagnosis details in Results section.

### Microbiological studies

The patient’s blood samples were incubated for five days using the BACTEC 9240 system (Becton–Dickinson Microbiology Systems, Franklin Lakes, NJ). Bronchoalveolar lavage (BAL) samples were cultured on blood agar, chocolate agar and Sabouraud dextrose agar with chloramphenicol and gentamicin (slant tube). Fungal isolates were identified by macroscopic and microscopic examination, as well as by MALDI-TOF MS and pan-fungal PCR targeting ITS region and sequencing.

Galactomannan antigen (GM) testing was performed using Platelia™ Aspergillus (Bio‐Rad Laboratories), with a cut‐off value of ≥ 0.5 in serum and ≥ 1.0 in BAL. Detection of 1,3‐β‐d‐glucan (BDG) in serum was performed with the Wako β‐glucan test (Fujifilm Wako Pure Chemical Corporation).

SRCD5CAR-NK-92 cells were subjected to exhaustive microbiological testing (including bacterial 16s rRNA, fungal 18S rRNA, and EBV, HCV, HBV and Mycoplasma DNA) by molecular (PCR and RT-PCR) and cell culture techniques available at the Microbiology Department of our center before infusion to the patient.

### Cell γ-irradiation

GFP/SRCD5CAR-NK-92 cells were **γ-**irradiated (10 Gy for 10min) immediately prior to infusion into the patient using a cesium irradiator (Mark 1-30; J.L Shepherd & Associates) at the School of Medicine of the Universitat de Barcelona.

### Immunological studies

HLA class I (A, B, and C) and II (DRB1 and DQB1) low resolution typing of the patient’s peripheral mononuclear cells and SRCD5CAR-NK-92 cells was performed by the PCR-SSOr (Sequence Specific Oligonucleotides reverse) technique using Lifecodes HLA-SSO typing kits (Immucor, Stamford, CT, USA).

KIR typing of SRCD5CAR-NK-92 cells was also performed by PCR-SSOr using the Lifecodes KIR-SSO typing kit (Immucor, Stamford, CT, USA).

Anti-HLA class I and II antibody monitoring pre and post SRCD5CAR-NK-92 infusion was performed by the Single Antigen bead assay using the Lifecodes LSA class I and class II kits (Immucor, Stamford, CT, USA).

Monitoring of the patient’s serum cytokine and chemokine levels was performed by Luminex^©^ technology using the bead-based Multiplex Assay HCYTOMAG-60K-16.Hu (Merck KGaA, Darmstadt, Germany) and following the manufacturer’s specifications.

Peripheral blood SRCD5CAR-NK-92 cells were monitored by flow cytometry by using the following monoclonal antibodies: anti-human CD56, anti-human CD34, anti-human CD5, anti-human CD337/NKp30, and anti-human CD16.

## Results

### Development and selection of NK-92 cells stably expressing the SRCD5CAR

**Figure 2:**
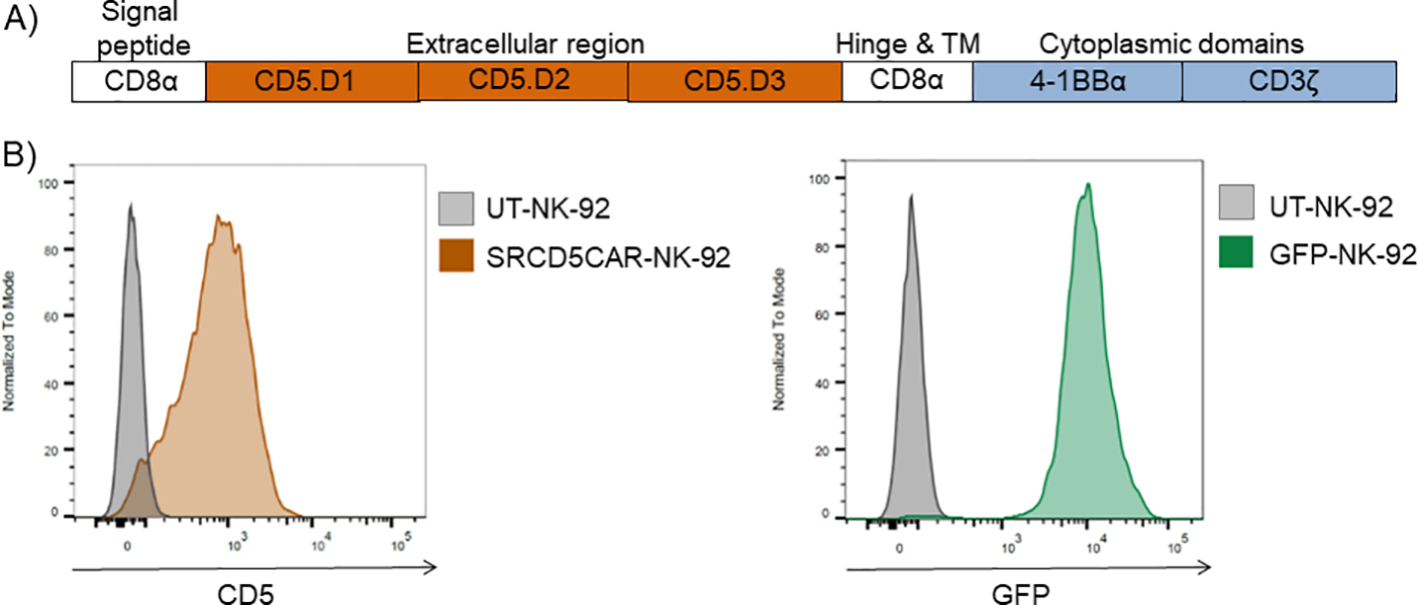
SRCD5CAR design and expression in lentivirally transduced NK-92 cells. **(A)** Schematic representation of the CD5CAR construct. **(B)** Flow cytometry analysis of CD5 (left) and GFP (right) expression in FACS selected SRCD5CAR-NK-92 and GFP-NK-92 cells, respectively. .

Building on the previously reported interaction between the human CD5 receptor and fungal β-glucans (20), it has been recently demonstrated that engineering primary (cord blood-derived) NK cells with a second-generation CD5-based CAR (SRCD5CAR) significantly enhances their innate, broad-spectrum antifungal activity, supporting their potential as a novel adoptive cell transfer therapy against IFI (19). Given that leukemia-derived NK-92 cells have also been reported to exhibit broad-spectrum antifungal activity (16, 17), our goal was to generate NK-92 cells stably expressing high levels of SRCD5CAR to further confirm their superior antifungal capabilities in functional *in vitro* and *in vivo* studies. To this end, NK-92 cells were transduced with lentiviral particles encoding the SRCD5CAR construct in which the prototypical single-chain variable fragment (scFv) was replaced by the whole ectodomain of human CD5 as fungal-recognition module. This was fused to the hinge and transmembrane regions of human CD8α and to the intracellular signaling domains of human CD28 and CD3ξ (**Figure 2A**). Following lentiviral transduction and subsequent immunomagnetic selection for surface CD5 positivity, we obtained a stable NK-92 cell population in which >95% of cells expressed SRCD5CAR (**Figure 2B**, left). For control purposes, a NK-92 cell population >95% positive for GFP expression was also generated by cell transfection and subsequent fluorescent-activated cell sorting (FACS) selection (**Figure 2B**, right).

### SRCD5CAR expression confers superior *in vitro* antifungal activity to NK-92 cells

**Figure 3:**
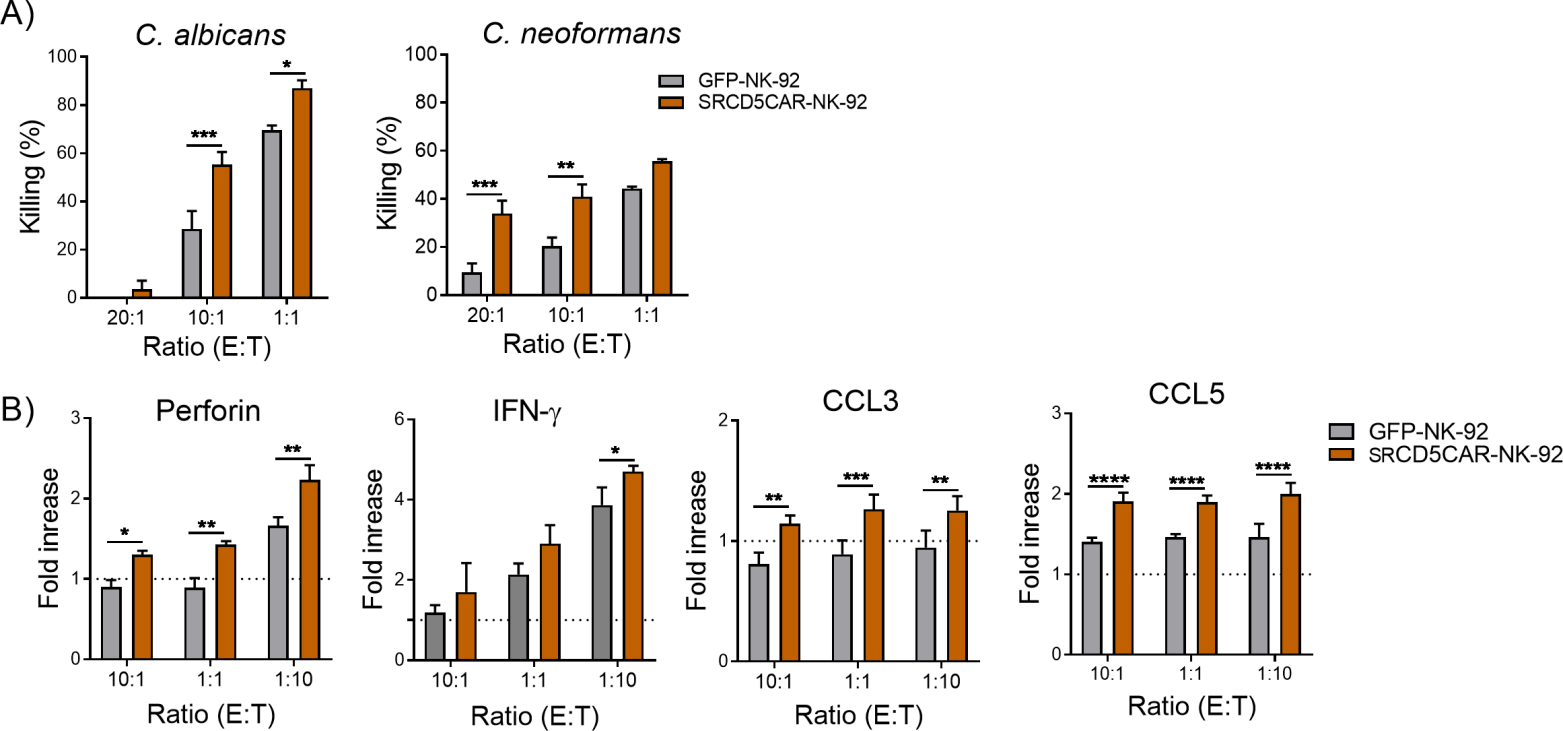
*In vitro* assessment of the antifungal activity of SRCD5CAR-NK-92 cells vs control GFP-NK-92 cells. **(A)** Fungal killing represented as percentage of CFU counts in supernatants from 2 h co-cultures of SRCD5CAR-NK-92 and GFP-NK-92 cells with live *C. albicans* (left) and *C. neoformans* (right) at the indicated E:T ratios. A representative experiment of three performed with results represented as mean ± SD (quadruplicate). **(B)** SRCD5CAR-NK-92 and GFP-NK-92 cells were co-cultured for 4 h with heat-killed *C. albicans* at the indicated E:T ratios. Culture supernatants were assayed for perforin, IFN-γ, CCL-3 and CCL-5 levels by ELISA. Representative experiment of three performed with results represented as mean ± SD (quadruplicates). Statistical differences were assessed by two-way ANOVA test (*, *p* < 0.05; **, *p* < 0.01; ***, *p* < 0.001; ****, *p* < 0.0001).

Previous *in vitro* studies demonstrate that NK-92 cells exhibit considerable damaging ability on different medically important fungal species (i.e, *Aspergillus*, *Candida*, mucormycetes, and *Fusarium*) (16, 17). In order to test whether SRCD5CAR expression confers NK-92 cells superior antifungal functionality, we performed *in vitro* killing assays in which SRCD5CAR-NK-92 and control GFP-NK-92 cells were co-cultured with *C. albicans* at different E:T ratios. As illustrated in **Figure 3A** left, SRCD5CAR-NK-92 cells displayed higher antifungal cytotoxicity than GFP-NK-92 controls at different E:T tested, as measured by the percentage of CFU counts with regard to fungal cells cultured alone. Similar results were obtained in co-cultures involving *C. neoformans* (**Figure 3A**, right),

NK-92 cell antifungal activity is exerted through both direct (e.g., exocytosis of lytic granules containing cytotoxic proteins) and indirect mechanisms (e.g., activation and mobilization of other immune cells via cytokine and chemokine release) (16, 17). Thus, we next tested the levels of selected soluble mediators in the supernatants of SRCD5CAR-NK-92 and GFP-NK-92 cells co-cultured with *C. albicans*. As illustrated in **Figure 3B**, SRCD5CAR-NK-92 cells secreted higher levels of cytotoxic proteins (i.e., perforin), and pro-inflammatory cytokines (i.e., IFN-γ) and chemokines (i.e., CCL3/MIP-1α and CCL5/RANTES) compared to GFP-NK-92 controls at the different E:T tested.

### SRCD5CAR expression confers superior *in vivo* antifungal activity to NK-92 cells

**Figure 4:**
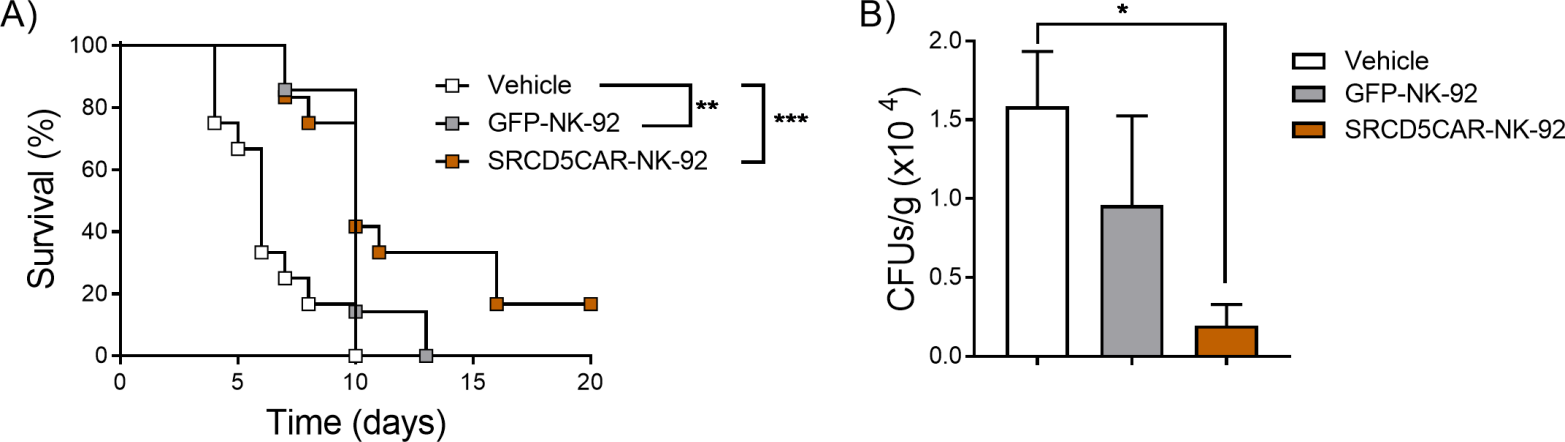
*In vivo* assessment of the antifungal activity of SRCD5CAR-NK-92 cells *vs*. control GFP-NK-92 cells. **(A)** Survival percentage of *C. albicans*-infected NSG mice (3 x 10^3^ CFU/mouse, *i.v.*) treated with a single *i.v.* infusion of SRCD5CAR-NK-92 (n=12), GFP-NK-92 (n=7) cells (2 x 10^6^ cells/mouse), or vehicle (n=12) at 24 h post-infection monitored over time (left). **(B)** In parallel experiments, kidney-fungal burden at 48 h post infection from NSG mice infected as in A and treated with a single *i.v.* infusion of SRCD5CAR-NK-92 (n=8), GFP-NK-92 (n=8) cells (2 x 10^6^ cells/mouse), or vehicle (n=8) at 24 h post-infection. Statistical differences were assessed by Log-rank (Mantel-Cox) or Kruskal-Wallis test (*, *p* < 0.05; **, *p* < 0.01; ***, *p* < 0.001).

The antifungal activity of SRCD5CAR-NK-92 cells was further assessed *in vivo* by their adoptive transfer to immunocompromised NSG mice undergoing systemic fungal infection. To this end, NSG mice *i.v.* infected with *C. albicans* (3 x 10^3^ CFU/mouse) received a single *i.v.* infusion of SRCD5CAR-NK-92 and GFP-NK-92 cells (2 x 10^6^ cells/mouse) or vehicle at 24 h post-infection. As illustrated in **Figure 4A**, mice treated with NK-92 cells – irrespective of SRCD5CAR- or GFP-expressing – exhibited significantly higher survival rates than the vehicle-treated group. Importantly, the SRCD5CAR-NK-92 treated group exhibited higher survival rates (16.6% *vs* 0% in both the GFP-NK-92 and vehicle-treated groups). Further confirmation of these results was obtained by fungal burden assessment in kidneys –the main target organ in the *C. albicans* infection model. As illustrated in **Figure 4B**, the SRCD5CAR-NK-92 treated group showed lower fungal burden levels than the GFP-NK-92 and vehicle control groups, as measured by CFU counts at 48 h post-infection.

### Compassionate use of SRCD5CAR-NK-92 cells in a case of multidrug-resistant fusariosis

**Figure 5:**
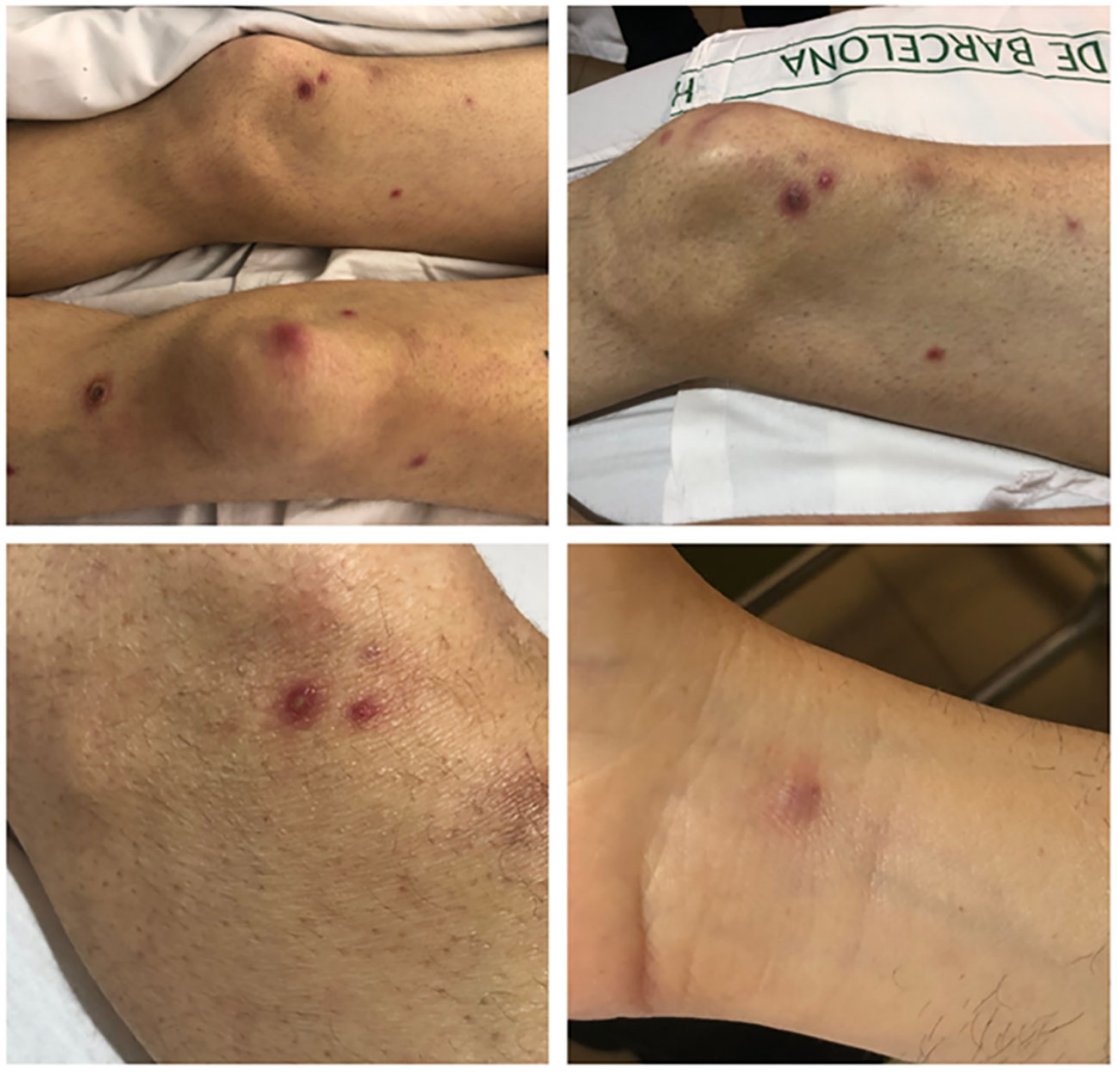
Initial skin lesions at hospital admission.

**Figure 6:**
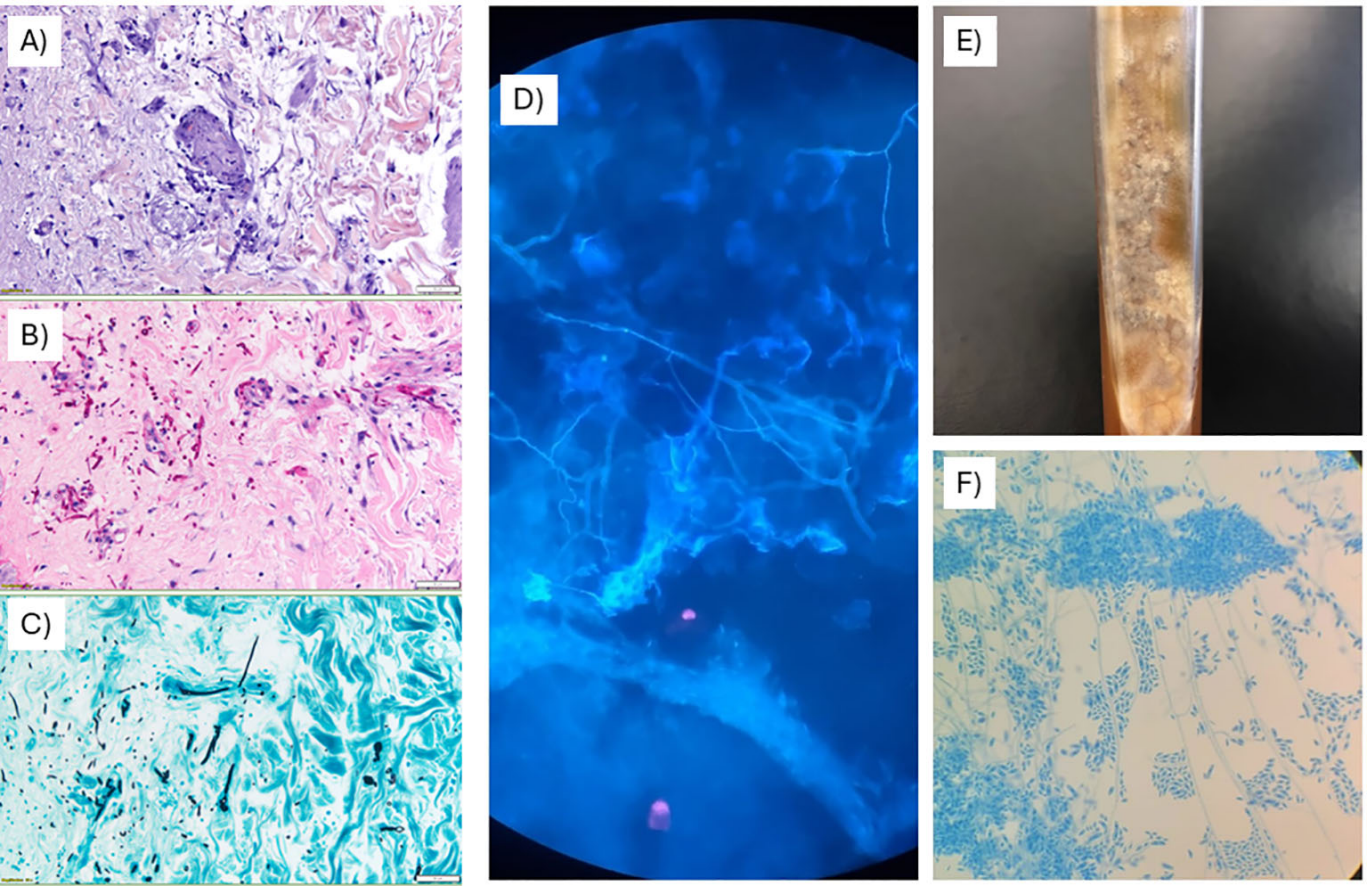
Anatomopathological and microbiological findings after skin biopsy. Left panels show anatomopathological samples (×20) with multiple fungal structures suggestive of mold infection on hematoxylin–eosin **(A)**, PAS–diastase **(B)**, and silver **(C)** stains. Right panels show direct visualization of mold hyphae with calcofluor white staining **(D)**, mold growth in culture **(E)**, and hyphae and conidia suggestive of *Fusarium* spp. on lactophenol cotton blue staining **(F)**. These results were obtained on day 3 after admission, seven days before SRCD5CAR-NK-92 administration.

While waiting for allo-HSCT, a patient was admitted with fever, myalgia, and the appearance of polytopic erythematous plaques and nodules on the trunk and extremities, some exhibiting central pustules (**Figure 5**). Blood tests confirmed known pancytopenia and a marked elevation of C-reactive protein (22 mg/dL). Blood cultures, galactomannan, and β-D-glucan tests produced negative results. Chest CT revealed multiple peribronchial solid nodules, predominantly localized in the right upper lobe (**Supplementary Figure 1** and **Supplementary Movie 1**). A bronchoscopy was performed but all microbiological results (including cultures and galactomannan testing) were negative. A skin biopsy was performed and microbiological and histochemical analyses confirmed a mold infection (**Figure 6**), with molecular identification of *Fusarium petroliphilum*. Antifungal susceptibility testing demonstrated high minimum inhibitory concentrations (MIC) for all antifungals tested: voriconazole and isavuconazole 8 mg/L; posaconazole and echinocandins >8 mg/L; and liposomal amphotericin-B at 2 mg/L. Upon admission, treatment with liposomal amphotericin B was initiated, and after the identification of *F. petroliphilum*, terbinafine and voriconazole were added to the therapeutic regimen. Despite these interventions, the patient remained febrile, with the development of new cutaneous lesions and mild worsening of the chest CT images. In this context, and based on pre-clinical antifungal efficacy data, compassionate use of SRCD5CAR-NK-92 cells therapy was considered.

**Figure 7:**
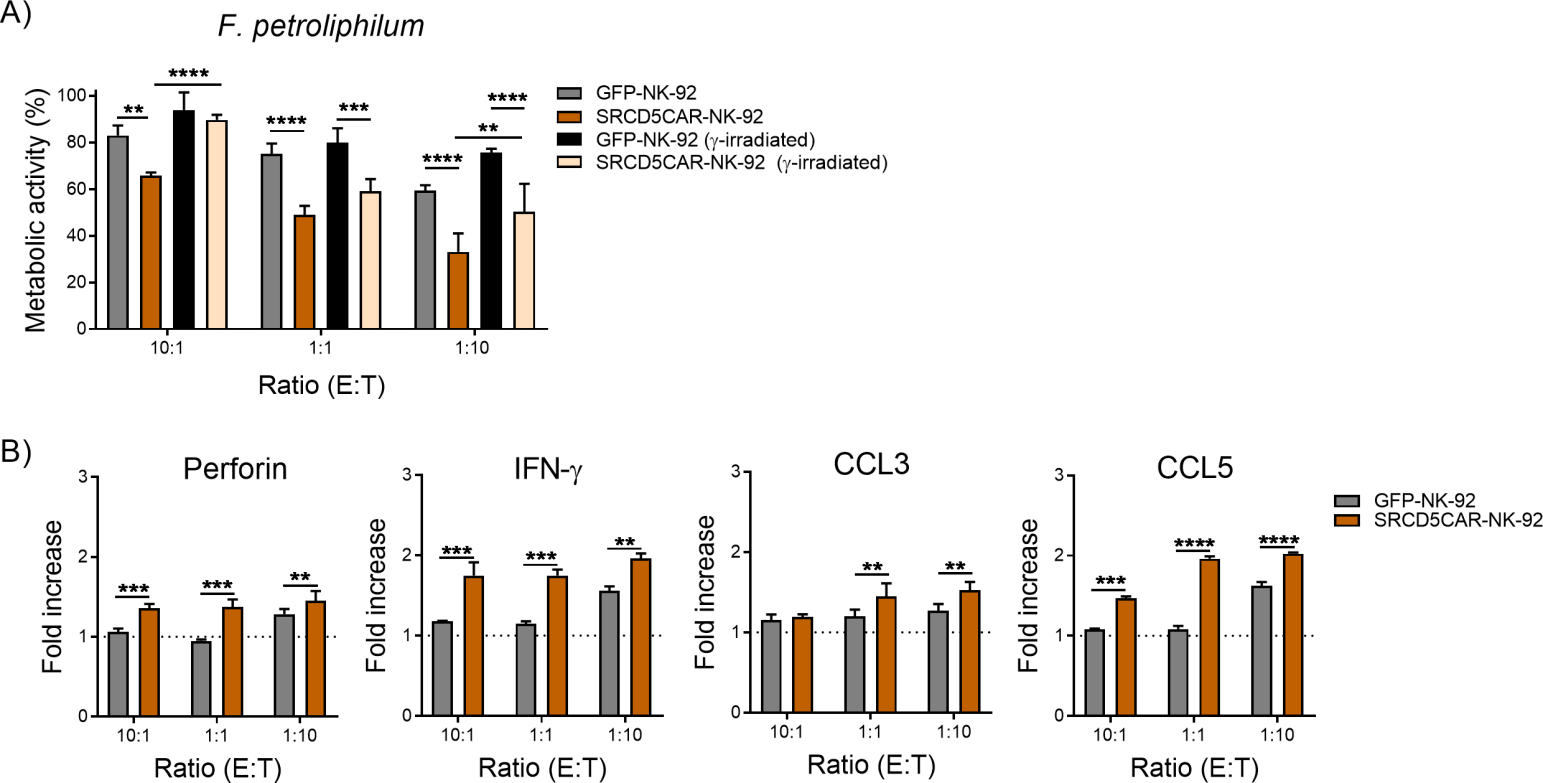
*In vitro* assessment of the antifungal activity of SRCD5CAR-NK-92 cells vs control GFP-NK-92 cells against the patient’s MDR *F. petroliphilum* isolate. **(A)** Percent of fungal metabolic activity as measured by XTT assays in 24 h co-cultures of γ-irradiated (10 Gy) and non-irradiated SRCD5CAR-NK-92 and GFP-NK-92 cells with live *F. petroliphilum* at the indicated E:T ratios. **(B)** SRCD5CAR-NK-92 and GFP-NK-92 cells were co-cultured for 24 h with heat-killed *F. petroliphilum* at the indicated E:T ratios. Culture supernatants were assayed for perforin, IFN-γ, CCL-3 and CCL-5 production by ELISA. Representative experiment of two performed with results represented as mean ± SD (quadruplicates). Statistical differences between groups were assessed by two-way ANOVA Test. (**, *p* < 0.01; ***, *p* < 0.001; ****, *p* < 0.0001).

Previous reports show that the broad antifungal activity of NK-92 cells also includes different *Fusarium* species (namely, *F. oxysporum*, *F. solani*, *F. proliferatum* and *F. verticillioides*) (17). So, it was hypothesized that this would also apply to the MDR *F. petroliphilum* isolate. This was confirmed by co-culturing the fungal isolate with GFP-NK-92 and SRCD5CAR-NK-92 cells at different E:T ratios, followed by assessment of fungal’s metabolic activity using XTT assays. As illustrated in **Figure 7A**, both GFP-NK-92 and SRCD5CAR-NK-92 cells induced lower *F. petroliphilum* metabolic activity in an E:T ratio-dependent manner. However, the antifungal activity of SRCD5CAR-NK-92 cells was consistently superior to that of GFP-NK-92 controls as deduced from the much lower metabolic activity of *F. petroliphilum*. Accordingly, the levels of perforin, IFN-γ, CCL3/MIP-1α and CCL5/RANTES in co-culture supernatants were also higher for SRCD5CAR-NK-92 cells compared to GFP-NK-92 controls (**Figure 7B**). In parallel *in vitro* experiments, the effects of γ-irradiation (10 Gy) on the antifungal activity of SRCRCD5-NK-92 and GFP-NK-92 cells against *F. petroliphilum* was also assessed. As illustrated in **Figure 7A**, compared to their non-irradiated counterparts γ-irradiation reduced the antifungal activity of both SRCD5CAR-NK-92 and GFP-NK-92 cells as deduced from higher metabolic activity values observed for *F. petroliphilum*. However, the antifungal activity of γ-irradiated SRCD5CAR-NK-92 cells was consistently superior to that of γ-irradiated GFP-NK-92 controls, and remained equivalent to that of non-irradiated GFP-NK-92 cells.

**Figure 8:**
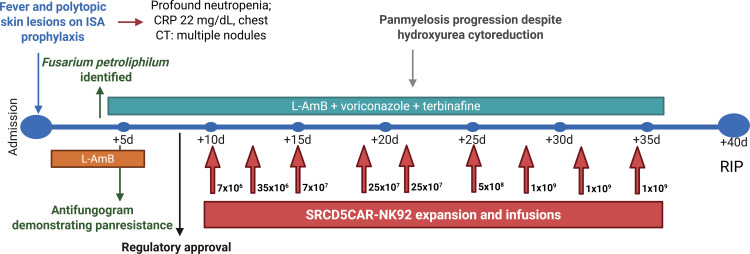
Summary of main patient’s findings and therapeutic interventions. CRP, C-reactive protein; CT, computed tomography; ISA, isavuconazole; L-AMB, liposomal amphotericin-B.

Following regulatory approval for a compassionate use and after a 72-h cell expansion process and exhaustive microbiological testing, SRCD5CAR-NK-92 cells were *i.v.* administered to the patient seven days after the initial *F. petroliphilum* isolation. For safety reasons, escalating doses of γ-irradiated (10 Gy) SRCD5CAR-NK-92 cells were administered at 2- to 5-day intervals (day 0, 7 x 10^6^ cells; day +2, 35 x 10^6^ cells; day +4, 7 x 10^7^ cells; days +9 and +11, 25 x 10^7^ cells; day +15, 5 x 10^8^ cells; days +18, +21, and +24, 1 x 10^9^ cells) (**Figure 8**).

### Response to the SRCD5CAR-NK-92 cell infusion

**Figure 9:**
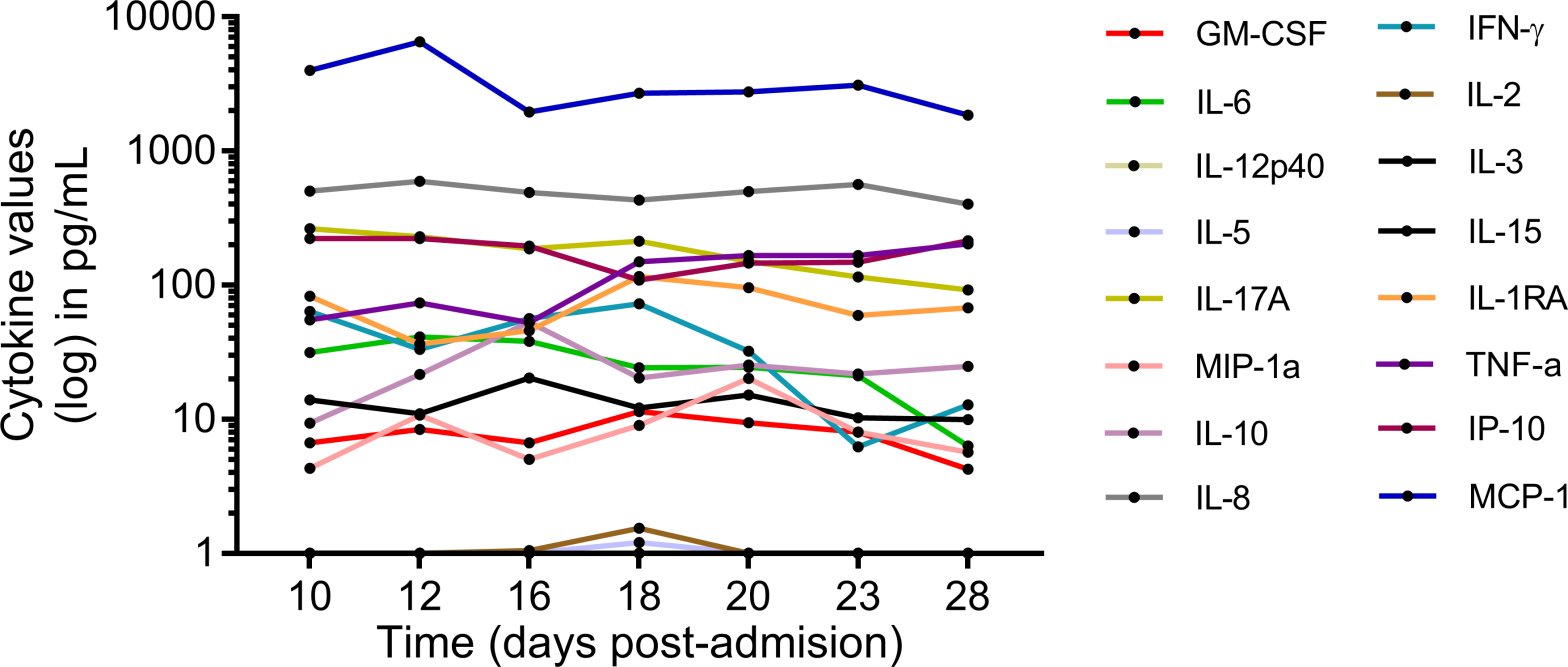
Patient’s serum cytokine levels over time.

No infusion-related or treatment-attributable adverse effects were documented following SRCD5CAR-NK-92 cell infusions. Mild cholestasis was noted, with gamma-glutamyl transferase and alkaline phosphatase levels reaching 679 U/L and 350 U/L, respectively, while transaminase and bilirubin levels remained within normal ranges. Kidney function and other biochemical parameters remained stable throughout therapy. Baseline pancytopenia persisted and showed no recovery during the hospital stay. Serial galactomannan monitoring was performed and remained consistently negative. No significant changes were detected in the levels of a broad panel of serum cytokines (IL-2, IL-3 IL-5, IL-6, IL-8, IL-10, IL-12p40, IL-15, IL-17A, GM-CSF, IFN-γ, TNF-α, IL-1ra) and chemokines (IP-10, MCP-1, MIP-1α) in serum samples collected just before cell infusions (**Figure 9**). Similarly, monitoring of anti-HLA class I and II antibodies pre- and post- infusion ofSRCD5CAR-NK-92 cells rendered negative results in spite of the several mismatches existing between SRCD5CAR-NK-92 and the patient’s HLA class I loci (**Supplementary Table 1**). Though KIR ligands HLA-A3/A11 and HLA-C1 were present in SRCD5CAR-NK-92 cells and absent from the patient, no evidence of alloreactivity through the missing self-activation mechanism was evidenced. In agreement with previous flow cytometric analyses of peripheral blood showing that irradiated NK-92 cells were undetectable immediately post infusion (28), we could not detect circulating SRCD5CAR-NK-92 cells (CD56++ CD5++ CD16- CD34- NKp30+) in blood samples taken just before infusions.

Throughout admission and following adoptive cell transfer, the patient developed persistent cough. No new skin lesions appeared and partial improvement of persisting ones was observed. Radiological findings from a follow-up chest CT performed 4 weeks after initiating SRCD5CAR-NK-92 cell infusions showed a mixed radiological evolution, with partial resolution of some pulmonary nodules and reduction in ground-glass opacities, alongside the emergence of new nodular infiltrates.

Unfortunately, while receiving the adjunctive SRCD5CAR-NK-92 cell infusions, the patient’s hematologic disease progressed with peripheral blood blasts increasing to 86%. Flow cytometry confirmed the expansion of blasts with the following immunophenotype: CD45+, CD34++, HLA-DR+, CD123+, CD117+, and TdT−, with expression of myeloid markers CD13+ and CD33 +. In addition, aberrant co-expression of CD56 (85%), CD5 (34%), and CD22 (67%) was observed. This profile is distinct from that of the infused NK-92 cells, which show CD56++, CD5++, CD34−, CD16−, and NKp30+. In the absence of further therapeutic options for his hematological malignancy, the treatment goal shifted to comfort care. The patient died on day +36 of admission after having received nine SRCD5CAR-NK-92 cell infusions. A necropsy was not authorized by the family.

## Discussion

The incidence of IFIs caused by multidrug-resistant (MDR) —both intrinsic or acquired— and aggressive fungi has progressively increased in recent years, following widespread use of antifungals (29–31). In this challenging scenario, the development of new therapeutic strategies is urgently needed. Based on our recent preclinical *in vitro* and *in vivo* data demonstrating the increased antifungal activity of primary NK cells engineered with a second generation CD5-based CAR (SRCD5CAR), the present work describe a similar performance of NK-92 cells stably expressing that SRCD5CAR against clinically relevant fungal species (i.e., *Candida*, *Cryptococcus* and *Fusarium*).

First evidence on antifungal adoptive cell therapy comes from a cohort of haplo-HSCT recipients with invasive aspergillosis (7). There, non-recipient reactive and *Aspergillus*-specific CD4^+^ T-cell clones were generated by co-culture of donor PBMC with heat-inactivated conidia, and further cytokine-expansion. Infected patients receiving such adoptive cell therapy in combination with amphotericin-B exhibited faster decrease in galactomannan levels and significantly lower mortality (10 *vs.* 46%) compared to the amphotericin-B sole group. Moreover, durable T-cell recovery suggested *in vivo* cell expansion (7). Despite these promising outcomes and the development of methods for generating GMP-grade fungal-specific CD4^+^ T-cells (32), HLA compatibility issues, along with intrinsic time and cost constraints, limited clinical implementation of this approach. A similar situation applies to antifungal adoptive CAR-T cell therapies currently in pre-clinical development (8, 10–12). Target recognition by CAR-T cells is not HLA-restricted, eliminating the need for antigen-presenting cells typically absent from pancytopenic patients, but these patients still express endogenous T-cell receptors (TCRs) prone to alloreactivity in non-autologous settings. In contrast, NK cells are naturally devoid of endogenous TCRs and exhibit minimal alloreactivity, thus reducing the risk of graft-vs-host disease (GVHD) and of high-intensity immune activation (i.e., cytokine release syndrome and immune effector cell-associated neurotoxicity syndrome), thus highlighting their potential as safe allogeneic off-the-shelf adoptive cell therapy (33). Moreover, NK cells exhibit antifungal activity, resulting directly from cytotoxicity via perforin and granzymes release, and indirectly from immune cell activation and recruitment via cytokine (e.g., INF-γ, TNF-α, and GM-CSF) and chemokine (e.g., CCL3/MIP-1a and CCL5/RANTES) release (16, 34). Accordingly, NK cells expanded *ex vivo* from PBMC have demonstrated potent Dectin-1-mediated fungicidal activity *in vitro* and reduced fungal burden in immunocompromised mice with pulmonary aspergillosis (35). Similarly, the leukemia-derived human NK-92 cell line, already extensively tested in cancer clinical trials (36), also exhibits killing activity against various medically significant fungal species, including *Aspergillus*, *Candida*, mucormycetes, and *Fusarium* (17). Unlike primary NK cells, NK-92 and other leukemia derived NK cell lines provide an unlimited, homogeneous, and low-cost source for adoptive cell transfer therapies, without the dependence on heterogeneous donors (37). In spite of their non-viability in the absence of exogenous IL-2 supply, an essential requirement for clinical application of NK cell lines is, however, irradiation prior to infusion, with doses that ensure proliferation arrest but cytotoxicity maintenance.

Building on this we aimed at developing NK-92 cells with enhanced *in vitro* and *in vivo* antifungal activity. This can be achieved by engineering them with conventional CARs in which the antigen-binding domain is an scFv targeting a fungal component, as has been recently reported for scFvk3-1-CAR-NK-92 cells against *C. albicans* mannans (18). In the SRCD5CAR-NK-92 cell case here presented, the scFv was replaced by the full-length extracellular region of the human lymphocyte scavenger receptor CD5. Several lines of evidence support the use of CD5 as fungal recognition domain: *i*) membrane-bound CD5 plays a non-redundant role in antifungal defense, as supported by the higher susceptibility of *Cd5*^-/-^ mice in experimental IFI models (38); *ii*) the extracellular region of human CD5 binds to fungal β-glucans with an affinity (*K*d) comparable to that of Dectin-1 (3.7 ± 0.2 nM *vs.* 2.6 mM–2.2 pM, respectively) (20, 38, 39); and *iii*) infusion of soluble human CD5 protein reduces mortality in mice subjected to zymosan-induced generalized inflammation (20), as well as undergoing *C. albicans* and *C. neoformans* infections (39). Importantly, the binding of CD5 to β-glucans ensures performance of SRCD5CAR-NK-92 against a broad spectrum of fungal species including MDR strains, since β-glucans are highly conserved, constitutive components of fungal cell walls not amenable to mutation without compromising viability and pathogenicity.

Following lentiviral transduction and further selection of NK-92 cells for stable and high surface expression of the second-generation SRCD5CAR, we provide evidence supporting superior *in vitro* and *in vivo* activity of SRCD5CAR-NK-92 *vs.* GFP-NK-92 control cells against different clinically relevant fungal species. Importantly, SRCD5CAR-NK-92 cells showed increased killing activity against a *F. petroliphilum* isolate resistant to available antifungals (voriconazole, isavuconazole, posaconazole, echinocandins, and liposomal amphotericin-B) from a patient with hematological malignancy. Given the limited antifungal therapeutic options available for this patient, a compassionate adjunctive treatment with SRCD5CAR-NK-92 cells was considered. Rapid and sufficient expansion of SRCD5CAR-NK-92 cells was achieved by *ex vivo* IL-2 supplementation, enabling successive and escalating *i.v.* cell infusions. For safety reasons, SRCD5CAR-NK-92 cells were γ-irradiated (10 Gy) before each infusion, an intervention known to prevent *in vivo* proliferation while preserving full cytotoxicity and cytokine production (36). To mitigate this limitation, together with the fact that NK-92 cell viability *in vivo* is reduced in the absence of exogenous IL-2 supplementation, repeated dosing every 2–5 days was implemented to maintain efficacy over time. Despite repeated infusions, induction of anti-HLA antibodies was not evidenced. This is in line with the moderate allogeneic response seen in previous phase I studies, in which only a subset of patients developed anti-HLA antibodies against NK-92 cells, all of which tested negative in mixed lymphocyte culture using patient lymphocytes as responder cells (25, 28).

In agreement with the previously reported antifungal activity of NK-92 cells against different *Fusarium* species (17), the *in vitro* killing activity of both SRCD5CAR-NK-92 and control GFP-NK-92 cells against the MDR *F. petroliphilum* isolate from the patient was confirmed. As expected, the *in vitro* antifungal activity was reduced by ~10-20% in both γ-irradiated SRCD5CAR-NK-92 and GFP-NK-92 cells. Of note, the antifungal activity of SRCD5CAR-NK-92 cells was consistently superior to GFP-NK-92 controls, irrespective of their previous γ-irradiation or not (**Figure 7A**).

Assessing the clinical impact of SRCD5CAR-NK-92 cell infusions proved challenging despite good tolerability. While no new skin lesions emerged, fever and cough persisted, and new pulmonary nodules appeared even if some showed favorable evolution. Several factors may have contributed to the lack of clear-cut efficacy, including reduced functionality of SRCD5CAR-NK-92 cells following γ-irradiation (lower γ-irradiation doses of 1–5 Gy could be considered), shortened half-life in the absence of exogenous IL-2 supply and, most importantly, insufficient cell dosage. Of the nine escalating infusions administered only the last three (1 x 10^9^ cells, roughly equivalent to 14 x 10^6^ cells/Kg for a patient weighing 70 Kg) approached the starting doses used in phase I clinical trials (1 x 10^9^ cells/m^2^) (36). This limitation may have hampered the efficacy of early infusions, resulting in incomplete regression of fungal lesions. Importantly, the rise in blast counts was consistent with progression of the underlying leukemia rather than a CAR-NK–related effect (40).

In summary, we report the first compassionate use of NK-92 cells expressing a CD5-based CAR as an adjunctive intervention in a case of MDR fusariosis. The lessons learned from this single patient case may help lay the foundation for future off-the-shelf clinical trials involving CD5CAR-engineered NK-92 cells for IFIs caused by a broad spectrum of pathogenic fungi, irrespective of their MDR status.

## Data Availability

The original contributions presented in the study are included in the article/**Supplementary Material**. Further inquiries can be directed to the corresponding authors.

## Supplementary material

Supplementary Table 1 HLA and KIR typing of patient and SRCD5CAR-NK-92 cells.

Supplementary Figure 1 Chest CT at admission. Dotted white circles highlight representative nodular lesions.

Supplementary Movie 1 Chest CT at admission. https://lluita-my.sharepoint.com/:v:/g/personal/ppuerta_lluita_org/EUSKzRcHREtFnWM1s2e1HHQBX7jbdT6U3bbRNOFrLztaaw?nav=eyJyZWZlcnJhbEluZm8iOnsicmVmZXJyYWxBcHAiOiJPbmVEcml2ZUZvckJ1c2luZXNzIiwicmVmZXJyYWxBcHBQbGF0Zm9ybSI6IldlYiIsInJlZmVycmFsTW9kZSI6InZpZXciLCJyZWZlcnJhbFZpZXciOiJNeUZpbGVzTGlua0NvcHkifX0&e=QvAZch.
