# Supplementary material for: Translational development and first-in-human compassionate infusion of NK-92 cells expressing a CD5-based chimeric antigen receptor (SRCD5CAR-NK-92) in a patient with multidrug-resistant fusariosis
Source: Front Immunol. 2026 May 8;17:1772830. doi: 10.3389/fimmu.2026.1772830 (PMC13194995; doi:10.3389/fimmu.2026.1772830)
Supplement: Supplementary Table 1 — HLA and KIR typing of patient and SRCD5CAR-NK-92 cells. [file DataSheet1.pdf]

Supplemental Information

Supplemental Table 1. HLA and KIR typing of patient and SRCD5CAR-NK92 cells.

	**Patient**	**SRCD5CAR-NK92**
**HLA class I typing**	A2, A24<br>B40, B44<br>Cw2, Cw4	A3, A11<br>B7, B44<br>Cw7, Cw16
**HLA class II typing**	DR7, DR16<br>DQ2, DQ5	DR7, DR15<br>DQ2, DQ6
**KIR typing**	N.D.	KIR2DL1, KIR2DL2, KIR2DL3, KIR2DL4<br>KIR2DS2, KIR2DS4<br>KIR3DL1, KIR3DL2, KIR3DL3<br>KIR2DP1, KIR3DP1

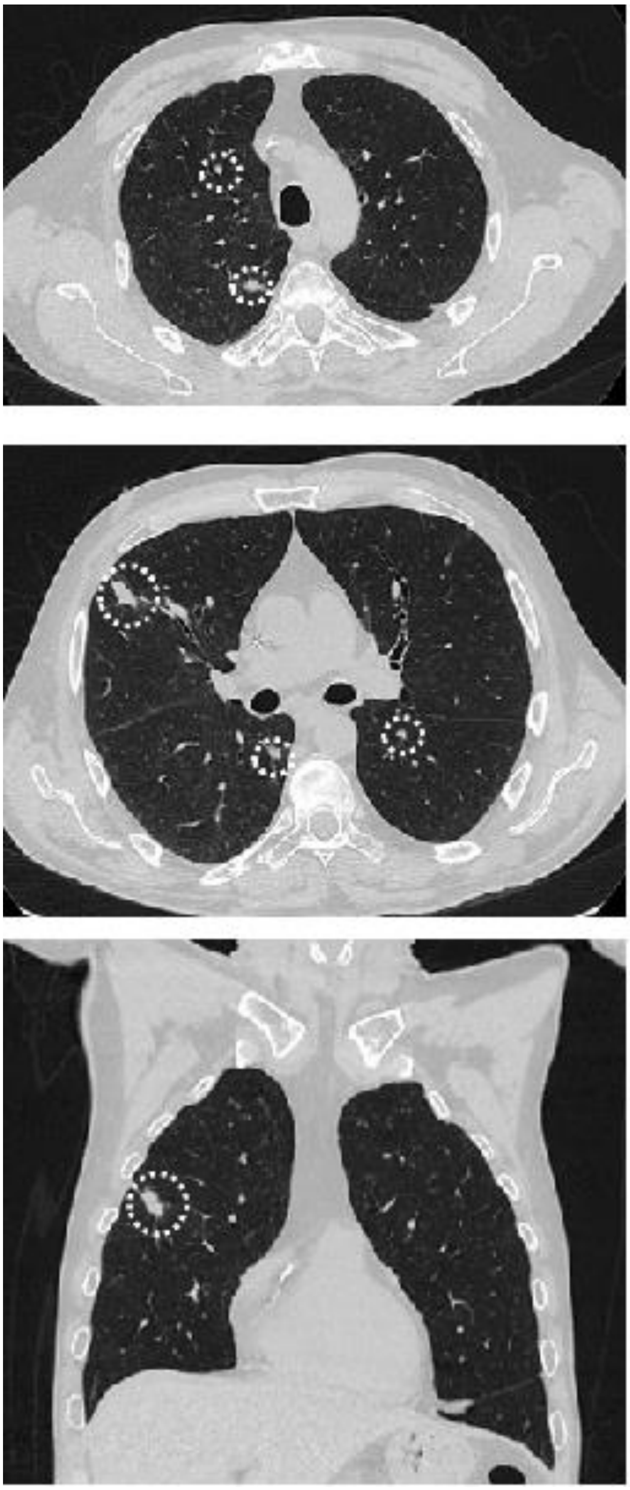

Supplemental Figure S1. Chest CT at admission. Dotted white circles highlight representative nodular lesions.

Supplemental Movie SM1. Chest CT at admission. https://lluita-my.sharepoint.com/:v:/g/personal/ppuerta_lluita_org/EUSKzRcHREtFnWM1s2e1HHQBX7jbdT6U3bbRNOFrLztaaw?nav=eyJyZWZlcnJhbEluZm8iOnsicmVmZXJyYWxBcHAiOiJPbmVEcml2ZUZvckJ1c2luZXNzIiwicmVmZXJyYWxBcHBQbGF0Zm9ybSI6IldlYiIsInJlZmVycmFsTW9kZSI6InZpZXciLCJyZWZlcnJhbFZpZXciOiJNeUZpbGVzTGlua0NvcHkifX0&e=QvAZch
